# Based on bioinformatics, SESN2 negatively regulates ferroptosis induced by ischemia reperfusion via the System Xc−/GPX4 pathway

**DOI:** 10.3389/fgene.2024.1504114

**Published:** 2025-01-29

**Authors:** Jiejie Hu, Lijun Qin, Guoqiang Zhu, Jingjing Ren, Hongxia Wang, Jing Jin, Haixue Zheng, Dan Li, Zhaoming Ge

**Affiliations:** ^1^ Department of Neurology, Lanzhou University Second Hospital, Lanzhou University, Lanzhou, China; ^2^ Department of Cardiology, Lanzhou University Second Hospital, Lanzhou University, Lanzhou, China; ^3^ School of Biological and Pharmaceutical Engineering, Lanzhou Jiaotong University, Lanzhou, China; ^4^ State Key Laboratory of Veterinary Etiological Biology, College of Veterinary Medicine, Lanzhou University, Lanzhou Veterinary Research Institute, Chinese Academy of Agricultural Sciences, Lanzhou, China; ^5^ Department of Internal Medicine, Gansu University of Traditional Chinese Medicine, Lanzhou, China

**Keywords:** ferroptosis, ischemia/reperfusion, brain microvascular endothelial cells, SESN2, system Xc-, RNA-seq, hub gene

## Abstract

**Introduction:**

Cerebral ischemia–reperfusion (IR) causes severe secondary brain injury. Previous studies have demonstrated that ferroptosis is involved in IR-induced brain injury. However, whether IR induces ferroptosis in brain microvascular endothelial cells (BMVECs) is not fully understood.

**Materials and methods:**

Oxygen–glucose deprivation/reoxygenation (OGDR) was performed in bEND.3 cells to mimic IR injury *in vitro*, and a focal cerebral IR model was created in C57BL/6 mice. Transcriptomic sequencing of the cells was performed first, followed by bioinformatics analysis. Differentially expressed gene (DEG) enrichment analysis highlighted ferroptosis-related pathways.

**Results:**

Using Venn analysis, nine ferroptosis-related DEGs were identified, namely, *Slc3a2*, *Slc7a11*, *Ccn2*, *Tfrc*, *Atf3*, *Chac1*, *Gch1*, *Lcn2*, and *Sesn2*. Protein–protein interaction (PPI) analysis combined with molecular complex detection (MCODE) identified six hub genes, namely, *Ddit3*, *Atf3*, *Sesn2*, *Trib3*, *Ppp1r15a*, and *Gadd45a*. Spearman’s correlation analysis revealed a significant correlation between the hub genes and ferroptosis-related DEGs. After reperfusion, the levels of ferroptosis indicators were elevated, and the expression of the ferroptosis-related proteins Xc− and GPX4 decreased. SESN2 is a hub gene and key antioxidant regulator. SESN2 silencing reduced the expression of System Xc− and GPX4, whereas overexpression of SESN2 promoted the expression of System Xc− and GPX4.

**Discussion:**

These results suggest that SESN2 is a negative regulator of ferroptosis. Enhancing the expression of SESN2 can alleviate ferroptosis through the activation of the System Xc−/GPX4 pathway. By integrating bioinformatics analysis with mechanistic exploration, this study revealed that ferroptosis plays a crucial role in IR-induced BMVECs injury, with SESN2 acting as a negative regulator via the System Xc−/GPX4 pathway.

## 1 Introduction

Stroke is the second highest cause of death globally, and ischemic stroke represents 71% of all strokes (Campbell and Khatri, 2020). Currently, endovascular reperfusion is the most effective treatment for acute ischemic stroke (Campbell and Khatri, 2020; Campbell et al., 2019). In addition to its benefits, blood reperfusion into the brain tissue accelerates brain cell death and tissue injury, known as cerebral ischemia-reperfusion (IR) injury, which is triggered by the inflammatory response, reactive oxygen species (ROS), and excitotoxicity (Li X. et al., 2021). Reperfusion injury often leads to the deterioration of brain function and predicts poor prognosis.

The breakdown of the blood–brain barrier (BBB), a natural barrier, is essential for maintaining central nervous system (CNS) homeostasis (Segarra et al., 2021). Brain microvascular endothelial cells (BMVECs) are the core components of the BBB and play an important role in its function and structural integrity. During reperfusion, BMVECs and BBB are severely damaged (Wang P. et al., 2022). Plasma-derived toxic substances infiltrate the brain parenchyma through the compromised BBB and exacerbate IR injury. Therefore, it is necessary to explore the mechanism of endothelial cell death after IR to protect the BBB and improve the prognosis.

Previous studies on brain IR injury have focused on oxidative stress (Zhang H. et al., 2024; He et al., 2020) and immune inflammation (Zhang H. et al., 2024; Zhou F. et al., 2021; Franke et al., 2021). In recent years, ferroptosis, a newly identified type of cell death, has been detected in brain IR injury; however, the role of ferroptosis in BMVECs death has not been well-explored.

Ferroptosis is a form of regulated cell death characterized by iron-dependent accumulation of lethal lipid hydroperoxides (Stockwell et al., 2017). Numerous biological processes are involved in ferroptosis, including amino acid, iron, and lipid metabolism and the biosynthesis of glutathione, phospholipids, and NADPH. The ferroptosis morphological manifestation (Costa et al., 2023) includes normal nuclei and shrinking mitochondria (membrane density increases, inner ridge reduces or disappears, and the outer membrane segment ruptures). The biochemical features of ferroptosis are characterized by iron accumulation, ROS accumulation, and lipid peroxidation, accompanied by System Xc− inhibition, GSH depletion, and GPX4 inactivation (Li X. et al., 2021; Costa et al., 2023).

Ferroptosis participates in the pathological process of multiple brain diseases, such as ischemic stroke (Cui et al., 2021; Li T. et al., 2023; Li J. et al., 2022; Zhu et al., 2022; Wang et al., 2023b), ischemia–reperfusion injury (Li T. et al., 2023; Zhu et al., 2022; Wang et al., 2023b; Wang et al., 2021), intracerebral hemorrhage (Li J. et al., 2022; Sun et al., 2022; Wan et al., 2019), traumatic brain injury (Li J. et al., 2022; Geng et al., 2021), and degenerative diseases (Costa et al., 2023; Li J. et al., 2022; Bao et al., 2021). Increasing evidence indicates that ferroptosis is a major contributor to cell death associated with IR injury (Li X. et al., 2021). Currently, studies on ferroptosis related to brain IR mainly focus on neurons and glial cells (Li T. et al., 2023; Wang et al., 2023b; Xu et al., 2023; Ryan et al., 2022). Some studies (Wang P. et al., 2022; Liu et al., 2023) have focused on the relationship between BMVECs and ferroptosis after reperfusion; however, this remains to be clarified.

SESN2 is a highly conserved, stress-inducing protein. Various stressors, such as hypoxia, oxidative stress, endoplasmic reticulum stress, inflammation, and autophagy (El-Horany et al., 2023; Zhang L. L. et al., 2024), upregulate SESN2 expression. As a key antioxidant modulator, SESN2 can relieve stress by decreasing ROS levels and inhibiting the mammalian target of rapamycin complex 1 (mTORC1) (Zhang L. L. et al., 2024). Previous studies have demonstrated that SESN2 induction efficiently ameliorates ferroptosis (El-Horany et al., 2023; Li J.-Y. et al., 2021; Yang et al., 2023; Yang et al., 2023) and exerts protective effects against IR injury in the intestine (Zhang L. L. et al., 2024), heart (Morrison et al., 2015), and brain (Yang et al., 2023).

In this study, we found that ferroptosis is an important death type of BMVECs after IR. Using RNA-seq combined with bioinformatic analysis, we identified nine ferroptosis-related DEGs and six hub genes. SESN2 is a hub gene. Given its well-established antioxidant effect, SESN2 is believed to have a profound association with ferroptosis. Thus, we further explored the regulatory role and underlying signaling pathways of SESN2 in ferroptosis through *in vitro* experiments. This study provides powerful theoretical support for further studies on BMVECs death and BBB disruption, following brain IR.

## 2 Materials and methods

### 2.1 Cell culture

bEND.3 cells were purchased from FuHeng Biology (catalog no. FH0356) and grown in T25 flasks (Corning) in DMEM supplemented with 10% fetal bovine serum at 37°C in a humidified normoxic incubator. Six cell samples were prepared for the RNA-seq experiment: three were used for normoxic culture (control group), and the remaining three were used for oxygen–glucose deprivation, followed by reoxygenation (OGDR group). bEND.3 cells for western blot and RT-qPCR were seeded onto 6-well plates and cultured as described above.

### 2.2 Oxygen–glucose deprivation/reoxygenation and re-glucose (OGDR)

When 90% confluence was reached, the bEND.3 cells were exposed to oxygen–glucose deprivation (OGD) for 10 h to mimic ischemia *in vitro*. In brief, bEND.3 cell growth complete medium was replaced with glucose-free DMEM (Solarbio, catalog no. D6540). Then, bEND.3 cells were placed in an anoxic incubator with 94% (v/v) nitrogen, 5% (v/v) carbon dioxide, and 1% (v/v) oxygen and incubated at 37°C. After 10 h, glucose-free DMEM was replaced with the normal growth complete medium, and bEND.3 cells were incubated for another 12 h at 37°C in a humidified normoxic incubator. To examine the expression trends of the protein GPX4, bEND.3 cells samples were harvested at 3, 6, 12, 24, and 36 h after reoxygenation and re-glucose supplementation. For the detection of mRNA level changes of Ccn2 and Tfrc, cells samples were also harvested at 3, 6, and 12 h following reoxygenation and re-glucose. For Fer-1 pretreatment, bEND.3 cells were incubated for 2 h with 20 μM Fer-1 before OGD.

### 2.3 Transcriptome sequencing

#### 2.3.1 RNA extraction

Total RNA was extracted from the cells using the TRIzol^®^ reagent, according to the manufacturer’s instructions. RNA quality was determined using a 5300 Bioanalyzer (Agilent, Santa Clara, CA, United States) and quantified using an ND-2000 instrument (NanoDrop Technologies). Only high-quality RNA sample (OD260/280 = 1.8 ∼ 2.2, OD260/230 ≥ 2.0, RIN ≥ 6.5, 28S:18S ≥ 1.0, and >1 μg) was used to construct the sequencing library.

#### 2.3.2 Library preparation and sequencing

RNA purification, reverse transcription, library construction, and sequencing were performed at Shanghai Majorbio Bio-pharm Biotechnology Co., Ltd. (Shanghai, China), according to the manufacturer’s instructions (Illumina, San Diego, CA, United States). The bEND.3 cell RNA-seq transcriptome library was prepared using 1 μg of total RNA, following the Illumina^®^ Stranded mRNA Prep, Ligation from Illumina (San Diego, CA). In brief, messenger RNA was isolated according to the polyA selection method using oligo (dT) beads and then initially fragmented using the fragmentation buffer. Double-stranded cDNA was synthesized using a SuperScript Double-Stranded cDNA Synthesis Kit (Invitrogen, Carlsbad, CA, United States) with random hexamer primers (Illumina). Then, the synthesized cDNA was subjected to end-repair, phosphorylation, and “A” base addition, according to Illumina’s library construction protocol. Libraries were selected for cDNA target fragments of 300 bp on 2% low-range ultra-agarose, followed by PCR amplification using Phusion DNA polymerase (NEB) for 15 PCR cycles. After quantification using Qubit 4.0, the paired-end RNA-seq library was sequenced using a NovaSeq X plus Sequencer (2 × 150 bp read length).

#### 2.3.3 Quality control and read mapping

Raw paired-end reads were trimmed, and quality was controlled using fastp with default parameters (Chen et al., 2018). Clean reads were then separately aligned to the reference genome in orientation mode using HISAT2 software (Kim et al., 2015). The mapped reads of each sample were assembled using StringTie (Pertea et al., 2015) with a reference-based approach. The batch effect of the data was evaluated by comparing visual PCA diagrams.

### 2.4 Bioinformatics

#### 2.4.1 Differential expression analysis

To identify DEGs between the control and OGDR groups, the expression level of each transcript was calculated according to the transcripts per million reads (TPM) method. RSEM (Li and Dewey, 2011) was used to quantify gene abundance. Differential expression analysis was performed using DESeq2 (Love et al., 2014). DEGs with |log2FC| ≥ 1 and P-adjust < 0.05 were considered to be significantly differentially expressed.

#### 2.4.2 Functional enrichment

In addition, functional enrichment analyses, including Gene Ontology (GO) and Kyoto Encyclopedia of Genes and Genomes (KEGG), were performed to identify which DEGs were significantly enriched in GO terms and metabolic pathways at a Bonferroni-corrected P-value < 0.05, compared to the whole-transcriptome background. GO functional enrichment and KEGG pathway analyses were performed using GoATools and Python scripts, respectively. Reactome functional enrichment was performed using a Python script. All data were analyzed using the online platform of Majorbio Cloud (https://cloud.majorbio.com/).

#### 2.4.3 Screening ferroptosis-related DEGs

Ferroptosis-related gene datasets were obtained from NCBI (https://www.ncbi.nlm.nih.gov/) and FerrDbV2 (http://www.zhounan.org/ferrdb/current/operations/download.html). After obtaining the dataset, a Venn analysis was performed to screen for DEGs associated with ferroptosis. DEGs with |log2FC| ≥ 1 and P-adjust < 0.05 were considered to be significantly differentially expressed.

#### 2.4.4 PPI analysis and molecular complex detection (MCODE)

The combined score was set to 0.4, and 207 DEGs between the two groups were used to construct the PPI network using the STRING database (version 11.5, https://string-db.org/). The PPI network was visualized using Cytoscape (v3.10.1). MCODE (degree cutoff: 2, node score cutoff: 0.2, k-core: 2, maximum depth: 100), an important plugin in Cytoscape, was used to screen the key sub-networks and hub genes.

#### 2.4.5 Correlation analysis

Spearman’s correlation analysis between hub genes and DEGs related to ferroptosis was performed using ChiPlot (https://www.chiplot.online/).

### 2.5 ROS, Fe^2+^, GSSG, and MDA assays

ROS levels in bEND.3 cells were measured using the Reactive Oxygen Species Assay Kit (Beyotime) in accordance with the manufacturer’s instructions. Simply, DCFH-DA was diluted with the serum-free culture medium to 1:1000 to make a final concentration of 10 μmol/L. bEND.3 cells were collected and suspended in diluted DCFH-DA at a concentration of 1 × 10^7^ cells/mL. After incubating for 20 min at 37°C, the cells were washed three times with the serum-free culture medium. EVOSTM M5000 (Invitrogen) was used to observe fluorescence signals in three random visual fields. ImageJ software was used to quantify the fluorescence signal intensity. Using Cell Ferrous Iron Colorimetric Assay Kits (E-BC-K881-M; Elabscience, China), relative Fe^2+^ concentrations in cell lysates were determined. The GSSG content in the cell lysates was measured using a GSSG assay kit (S0053; Beyotime, China). The MDA content in the cell lysates was detected using an MDA assay kit (S0131S; Beyotime, China). The kits were used according to the manufacturer’s instructions.

### 2.6 Transmission electron microscopy (TEM)

Mitochondrial morphology was examined using TEM. bEND.3 cells in the control and OGDR groups were fixed in 0.1 M phosphate buffer with 2.5% glutaraldehyde (Merck) for 1 h at 25°C, post-fixed with 2% osmium tetroxide, and embedded in epoxy resin. After polymerization, sections (80 nm thick) were obtained and stained with uranyl acetate and lead citrate. The samples were observed using a transmission electron microscope (Hitachi, HT7700, Japan).

### 2.7 Mouse model of brain ischemia–reperfusion

Male C57BL/6 mice, 6–8 weeks old and pathogen-free were purchased from the Lanzhou Veterinary Research Institute (Lanzhou, China). Mice were kept in a temperature- and humidity-controlled animal facility with a 12 h light-dark cycle. Food and water were provided *ad libitum*. Twenty mice were used in the present study. All procedures were approved by the Institutional Ethics Committee of the Lanzhou Veterinary Research Institute.

To establish a focal cerebral ischemia model, a 7-0 suture (Doccol Corporation) was introduced at the origin of the middle cerebral artery through the right common carotid artery to block the blood flow. Reperfusion was performed by removing the sutures. The animals were allowed 30 min of blood blockade, followed by 24 h of reperfusion. During the ischemic period, body temperature was maintained at 37.0°C ± 0.5°C by a temperature-controlled heating pad.

Mice were deeply anesthetized with isoflurane and transcardially perfused with ice-cold saline, followed by 4% paraformaldehyde (PFA). Brains were harvested, post-fixed in 4% PFA, and embedded in paraffin. Serial coronal brain sections at a thickness of 3 μm were prepared for histochemical analysis and immunofluorescence.

### 2.8 Histochemical analyses

DAB-enhanced Perls’ Prussian blue was used to evaluate the iron load in the brain tissue. According to the manufacturer’s protocol, the sections were placed in a Prussian blue solution (Servicebio, Wuhan, China) for 30 min and then stained with the DAB solution (Servicebio, Wuhan, China) for 10 min. Sections were digitized using a DS-U3 Digital Camera (Nikon, Japan).

### 2.9 Immunofluorescence staining

For immunofluorescence, after deparaffinization, rehydration, and antigen repair, the brain sections were blocked with 3% BSA for 30 min at room temperature and then incubated overnight with the following primary antibodies: rabbit anti-SESN2 (1:100 dilution, Abcam, catalog no. ab178518, UK), rabbit anti-HMOX1 (1:600; Servicebio, catalog no. GB11549-50, China), rabbit anti-SLC7A11 (1:1000; Abcam, UK; catalog no. ab307601, UK), rabbit anti-SLC3A2 (1:1000; Abcam, catalog no. ab303510, UK), rabbit anti-GPX4 (1:1000 dilution; Thermo Fisher Scientific, catalog no. PA5-109274, United States), or mouse anti-CD31 (1:500 dilution; catalog no. GB120005-50, China). After washing, sections were incubated with secondary antibodies conjugated to Alexa Fluor 488 (1:400 dilution; catalog no. GB25303, China) and Cy3 (1:300 dilution; catalog no. GB21301; China) for 1 h at room temperature, and the nuclei were counterstained with DAPI. Sections were incubated with all solutions except for the primary antibodies to serve as negative controls for assessing nonspecific signals. Images were acquired using Pannoramic MIDI (3DHISTECH).

For *in vitro* experiments, bEND.3 cells were seeded in a laser confocal dish (Corning), fixed with 4% PFA, and permeabilized with 0.2% Triton X-100. The fixed cells were blocked with 5% BSA for 1 h and incubated overnight with the following primary antibodies: rabbit anti-SESN2 (1:100 dilution, Abcam, catalog no. ab178518, UK), rabbit anti-SLC7A11 (1:500; Abcam, catalog no. ab307601, UK), rabbit anti-SLC3A2 (1:50; Abcam, catalog no. ab303510, UK), or rabbit anti-GPX4 (1:50 dilution, Thermo Fisher Scientific, catalog no. PA5-109274, United States). The cells were then washed with PBS, incubated with secondary antibodies conjugated to Alexa Fluor 488 (1:500 dilution, CST, catalog no.4412S, United States) for 1 h, and counterstained with DAPI. Images were acquired using a laser confocal microscope (LSM 980, Airyscan 2).

### 2.10 Transfection

bEND.3 cells were transfected with specific or negative control siRNAs (Tsingke Biotech, Beijing, China). The plasmids were purchased from Tsingke Biotech (Beijing, China). Transfections were performed using jetPRIME® *in vitro* DNA and siRNA transfection reagent (114-15, Polyplus, France) reagent for 6 h. The cells were collected 48 h after transfection for western blotting and RT-qPCR analyses.

### 2.11 Western blot

Brain tissues or cells were lysed using the RIPA lysis buffer (PC102; Epizyme, China) supplemented with protease inhibitors (GRF101; Epizyme, China) and phosphatase inhibitors (GRF101; Epizyme, China). The samples were ultrasonically broken on ice, and protein quantification was performed using a BCA protein assay kit (P0009, Beyotime, China). Protein samples were then mixed with the loading buffer (LT103, Epizyme, China) and heated for 10 min at 100°C. Total protein was separated by SDS-PAGE and transferred to the NC membrane (Pall). The electrophoresis conditions were as follows: 10% gel, 25°C, 150 V, and 60 min; the transfer conditions were as follows: ice bath, 100 V, and 60 min. Transferred membranes were blocked with 5% skim milk for 1 h at 25°C and incubated overnight at 4°C with the primary antibodies: rabbit anti-SESN2 (1:1000 dilution, Abcam, catalog no. ab178518, UK), rabbit anti-SLC7A11 (1:1000; Abcam, catalog no. ab307601, UK), rabbit anti-SLC3A2 (1:1000; Abcam, catalog no. ab303510, UK), rabbit anti-GPX4 (1:1000 dilution; Thermo Fisher Scientific, catalog no. PA5-109274, United States), or β-actin (1:1000 dilution; catalog no. A5441, United States). Then, membranes were incubated with horseradish peroxidase-conjugated secondary antibodies (1:5,000 dilution, Santa, United States) for 1 h at 25°C. Proteins were detected using the Pierce ECL western Blotting Substrate (Thermo Fisher Scientific) in the dark using a gel imaging system (Bio-Rad, United States). ImageJ software was used to quantify the western blot signals. The relative protein amounts were corrected by β-actin.

### 2.12 RT-qPCR

Total cellular RNA was isolated using the TRIzol reagent (Invitrogen) from the cultured cells, and then, RNA was transcribed into cDNA using a reverse transcription kit (PrimeScript™ RT Master Mix, Takara, Code No. RR047A). For reverse transcription, the following system was used: 2 μL of 5× PrimeScript RT Master Mix and 1 μg of RNA supplemented with DEPC-treated water to a final volume of 10 μL. The reaction conditions were as follows: 37°C for 30 min, followed by 85°C for 5 s. Afterward, cDNA was stored at −80°C for subsequent detection. Transcription levels of targeted genes were assessed by RT-qPCR analysis using TB Green^®^ Premix Ex Taq™ II (TaKaRa, Code No. RR820A). The reaction system was as follows: 7 μL of TB Green^®^ Premix Ex Taq™ II, 1 μL of upstream primes, 1 μL of downstream primes, and 5 μL of diluted cDNA (200×), and the reaction procedure was as follows: 95°C for 3 min, followed by 95°C for 3 s, and 60°C for 10 s, for a total of 40 cycles. Three replicates were performed for each experiment. Gene expression levels were normalized to *GAPDH*. Relative mRNA expression levels were calculated utilizing the 2^−ΔΔCT^ method and normalized to the control group. All the primers used in the RT-qPCR assays are listed in Table 1.

**TABLE 1:** Information on primers for RT-qPCR amplification.

Gene (*Mus*)	Forward	Reverse
*Ddit3*	CTG​GAA​GCC​TGG​TAT​GAG​GAT	CAG​GGT​CAA​GAG​TAG​TGA​AGG​T
*Atf3*	GAG​GAT​TTT​GCT​AAC​CTG​ACA​CC	TTG​ACG​GTA​ACT​GAC​TCC​AGC
*Sesn2*	TCC​GAG​TGC​CAT​TCC​GAG​AT	TCC​GGG​TGT​AGA​CCC​ATC​AC
*Trib3*	GCA​AAG​CGG​CTG​ATG​TCT​G	AGA​GTC​GTG​GAA​TGG​GTA​TCT​G
*Gadd45a*	CCG​AAA​GGA​TGG​ACA​CGG​TG	TTA​TCG​GGG​TCT​ACG​TTG​AGC
*Ppp1r15a*	GAG​GGA​CGC​CCA​CAA​CTT​C	TTA​CCA​GAG​ACA​GGG​GTA​GGT
*Slc3a2*	TGA​TGA​ATG​CAC​CCT​TGT​ACT​TG	GCT​CCC​CAG​TGA​AAG​TGG​A
*Slc7a11*	TGG​GTG​GAA​CTG​CTC​GTA​AT	AGG​ATG​TAG​CGT​CCA​AAT​GC
*Tfrc*	GTT​TCT​GCC​AGC​CCC​TTA​TTA​T	GCA​AGG​AAA​GGA​TAT​GCA​GCA
*Ccn2*	GGG​CCT​CTT​CTG​CGA​TTT​C	ATC​CAG​GCA​AGT​GCA​TTG​GTA
*Lcn2*	TGG​CCC​TGA​GTG​TCA​TGT​G	CTC​TTG​TAG​CTC​ATA​GAT​GGT​GC
*Chac1*	CTG​TGG​ATT​TTC​GGG​TAC​GG	CCC​CTA​TGG​AAG​GTG​TCT​CC
*Gch1*	ACT​TCA​CCA​AGG​GAT​ACC​AGG	CTT​GCT​TGT​TAG​GAA​GAT​AGC​CA
*Gpx4*	GAT​GGA​GCC​CAT​TCC​TGA​ACC	CCC​TGT​ACT​TAT​CCA​GGC​AGA
*Gapdh*	AGG​TCG​GTG​TGA​ACG​GAT​TTG	TGT​AGA​CCA​TGT​AGT​TGA​GGT​CA

### 2.13 Statistical analyses

Statistical analyses were conducted using GraphPad Prism 9 (Version 9.5.1 (733), GraphPad Software). Data were displayed as the mean ± SD. The unpaired two-tailed Student’s t-test was used for comparison between the two groups. Statistical significance was set at p < 0.05. Spearman’s correlation analysis was used to analyze the correlation between the two datasets.

## 3 Results

### 3.1 Sample preparation and RNA-sequencing data quality control

**FIGURE 1:**
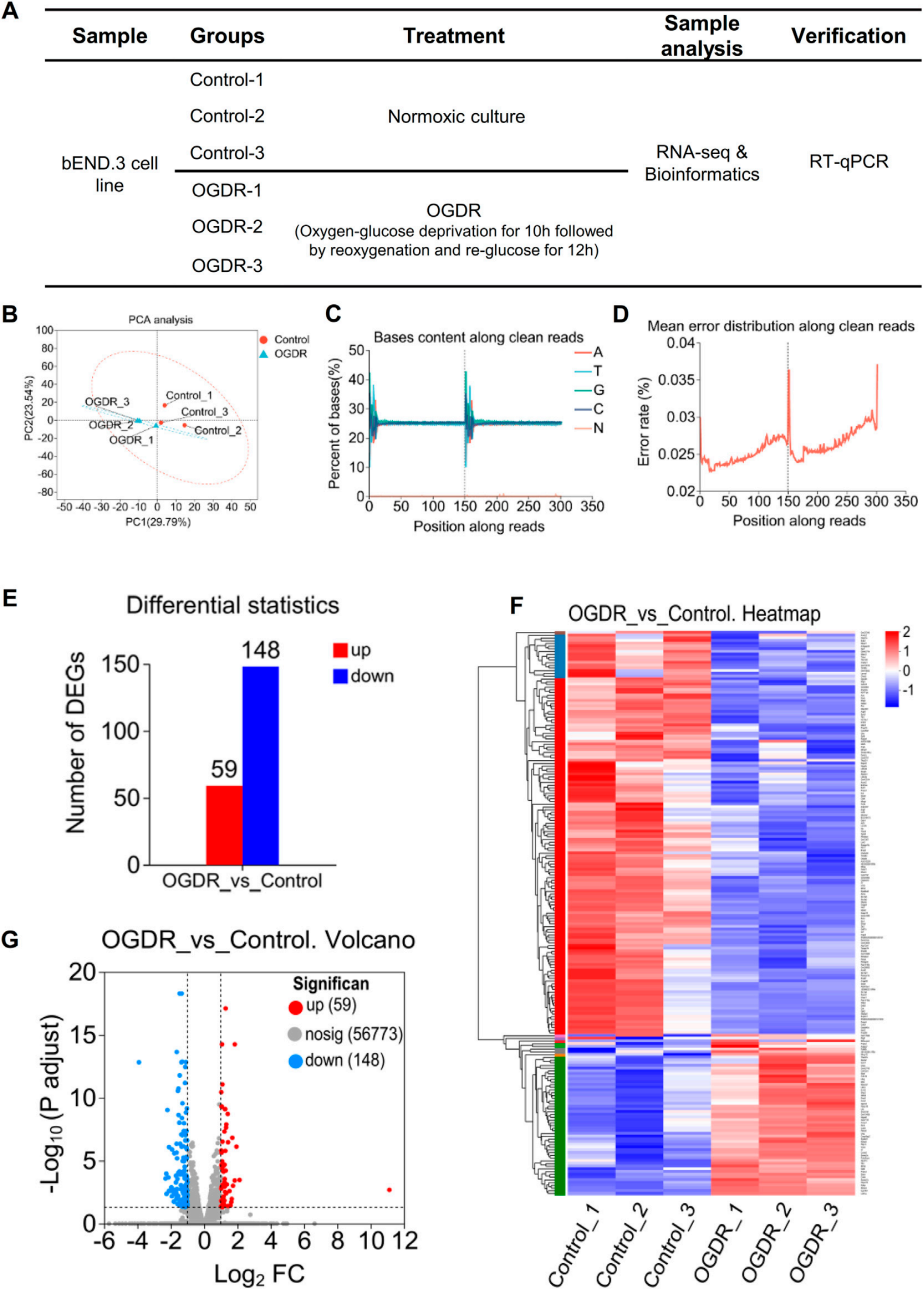
RNA-seq and obtaining DEGs. **(A)** Sample design diagram for RNA-seq. **(B–D)** Visualization of RNA-seq data quality control: PCA analysis between samples **(B)**, bases content along clean reads **(C)**, and mean error distribution along clean reads **(D)**. **(E–G)** DEGs in the OGDR group compared to the control group. Based on the quantitative results of the expression level, the differential gene analysis was carried out to obtain DEGs, the difference analysis software used was DESeq2, and the screening threshold was |log2FC| ≥ 1 and p-adjust < 0.05; p-value multiple test correction method: BH. **(E)** Histogram of DEGs: the red bar represents the upregulated genes and blue bar for the downregulated genes. **(F)** Heatmap of DEGs. **(G)** Volcano plot of DEGs: the red and blue plots refer to upregulated and downregulated genes, respectively.

To study the damage to brain microvascular endothelial cells during IR, we cultured bEND.3 cells *in vitro* and implemented OGDR treatment. Cell samples from the control and OGDR groups were collected and subjected to RNA-seq (Figure 1A). We performed data quality control to ensure the library construction and sequencing quality of the samples. The base content and base error rate of each sample were analyzed (Figures 1C, D), and the alignment rate of the reads to the reference genome met the requirements (Table 2). The base error rates (0.0255%–0.026%), Q20 (97.03%–97.29%), Q30 (94.67%–95.13%), and GC content (50.59%–51.51%) met these requirements (Table 3). PCA showed that the biological reproducibility of the samples met the requirements and that the reproducibility of the samples in the OGDR group was better than that in the control group (Figure 1B).

**TABLE 2:** Comparative analysis with a reference genome.

Sample	Total reads	Total mapped	Multiple mapped	Unique mapped
Control_1	45,327,944	43,633,657 (96.26%)	2,470,997 (5.45%)	41,162,660 (90.81%)
Control_2	45,402,770	43,690,672 (96.23%)	2,531,854 (5.58%)	41,158,818 (90.65%)
Control_3	43,912,048	42,267,846 (96.26%)	2,342,002 (5.33%)	39,925,844 (90.92%)
OGDR_1	49,954,600	48,182,629 (96.45%)	2,964,763 (5.93%)	45,217,866 (90.52%)
OGDR_2	44,279,084	42,527,787 (96.04%)	2,337,197 (5.28%)	40,190,590 (90.77%)
OGDR_3	5,3,791,708	51,829,837 (96.35%)	3,139,791 (5.84%)	48,690,046 (90.52%)

Reference gene source: *Mus musculus*; reference genome version: GRCm39; reference genomic source: http://asia.ensembl.org/Mus_musculus/Info/Index. The clean reads of each sample were sequence-aligned with *Mus musculus* genome. Matching rates: 96.04%–96.75%.

**TABLE 3:** RNA sequencing data statistics analysis.

Sample	Raw reads	Raw bases	Clean reads	Clean bases	Error rate (%)	Q20 (%)	Q30 (%)	GC content (%)
Control_1	45,941,938	6,937,232,638	45,327,944	6,619,169,433	0.0258	97.11	94.83	50.59
Control_2	46,000,084	6,946,012,684	45,402,770	6,640,948,081	0.026	97.03	94.69	51.51
Control_3	44,449,978	6,711,946,678	43,912,048	6,433,724,546	0.0259	97.07	94.76	51.28
OGDR_1	50,441,644	7,616,688,244	49,954,600	7,292,255,355	0.0255	97.29	95.13	50.81
OGDR_2	44,798,362	6,764,552,662	44,279,084	6,506,145,580	0.026	97.03	94.67	50.69
OGDR_3	54,388,116	8,212,605,516	5,3,791,708	7,816,683,677	0.0255	97.27	95.1	50.65

### 3.2 DEG function analysis revealed that ferroptosis may play an important role in the OGDR injury

In order to obtain significantly differentially expressed genes, we used DESeq2 software to analyze RNA-sequencing data with the following parameter criteria: |log2FC| ≥ 1 and p-adjust < 0.05. There were 207 DEGs in the OGDR group compared with the control group, including 59 upregulated and 148 downregulated genes (Figure 1E). The heatmap analysis and volcano plot were used to visualize the results of the difference analysis (Figures 1F, G).

**FIGURE 2:**
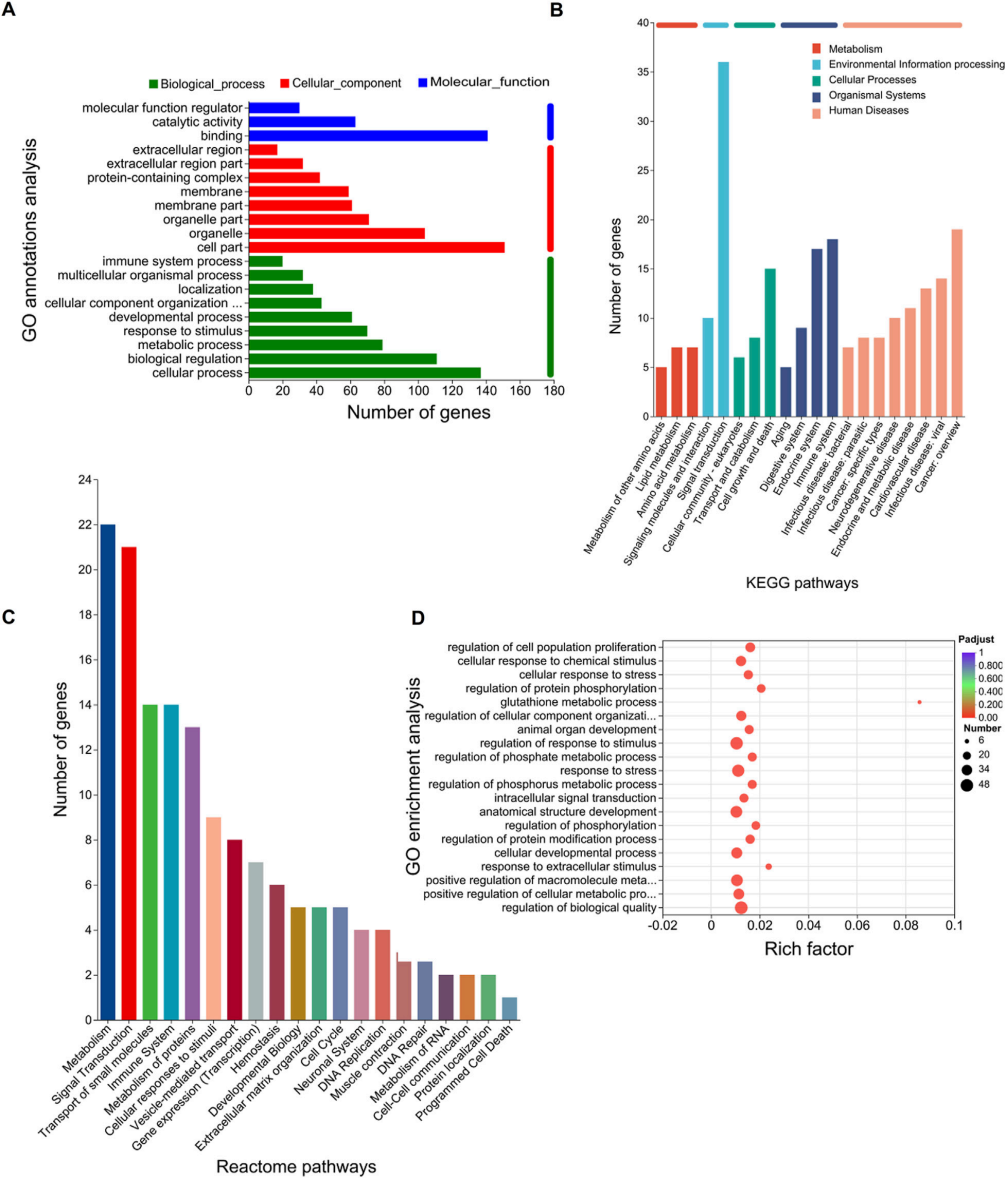
Functional enrichment analysis of DEGs. The bar chart length represents the number of genes. **(A)** GO annotation analysis. The top 20 terms in abundance are shown, including 9 terms in BP, 8 terms in CC, and 3 terms in MF. **(B)** KEGG pathways. The top 20 KEGG pathways in abundance are shown. **(C)** Reactome pathways. The top 20 Reactome pathways in abundance are shown. **(D)** GO enrichment analysis. Screening threshold for significant enrichment, p-adjust ≤ 0.05. The top 20 enrichment terms in abundance are shown. The higher the rich factor, the more significant the enrichment. The sizes of the dots indicate the number of genes/transcripts in this GO term, and the color of the dot corresponds to different p-adjust ranges. The GO term with the most significant enrichment is the glutathione metabolic process (GO:0006749, rich factor: 0.0857142857143, term type: BP, p-adjust: 0.00109589801972).

To further explore the functions of the 207 DEGs, GO, KEGG, and Reactome pathway analyses were conducted. On GO annotation analysis, for the biological process (BP), cell component (CC), and molecular function (MF) categories, DEGs were mainly enriched in “cellular process,” “biological regulation,” and “metabolic process” in the BP category; “cell part,” “organelle,” and “organelle part” in the CC category; and “binding,” “catalytic activity,” and “molecular function regulator” in the MF category (Figure 2A). In KEGG pathway analysis, DEGs were mainly enriched in “amino acid metabolism” and “lipid metabolism” in “metabolism” and “cell growth and death” in “cellular process” (Figure 2B). In Reactome pathway analysis, the DEGs were mainly enriched in “metabolism” and “signal transductions” (Figure 2C). In GO enrichment analysis, the term with the largest rich factor is “glutathione metabolic process” (Figure 2D). The main enriched pathways were associated with amino acid metabolism, lipid metabolism, glutathione metabolism, and cell growth and death. The results of the functional analysis suggested that ferroptosis might occur in the OGDR group.

### 3.3 Nine ferroptosis-related DEGs and six hub genes were obtained, and the verification was performed by RT-qPCR

**FIGURE 3:**
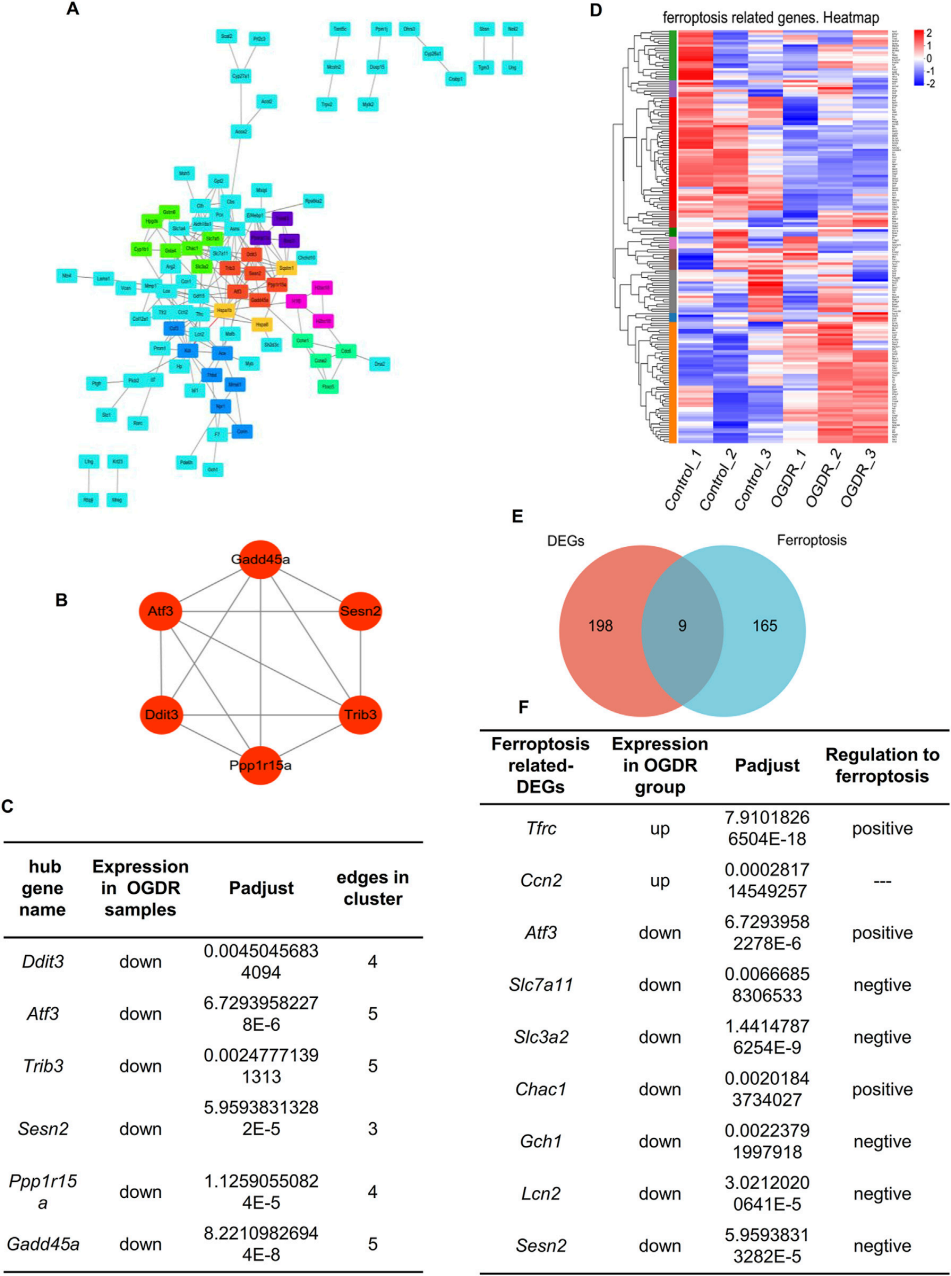
Hub genes and ferroptosis-related DEGs. **(A)** Visual diagram of 207 DEG PPI network using Cytoscape, including 113 nodes and 207 edges. Screening out seven clusters and different colors for different clusters. Red for cluster 1 (6 genes, score: 5.200), light green for cluster 2 (7 genes, score: 4.000), dark green for cluster 3 (4 genes, score: 4.000), blue for cluster 4 (7 genes, score: 3.333), purple for cluster 5 (3 genes, score: 3.000), pink for cluster 6 (3 genes, score: 3.000), and orange for cluster 7 (3 genes, score: 3.000). Cluster 1 gets the highest score, and six genes (*Ddit3*, *Atf3*, *Sesn2*, *Trib3*, *Gadd45a*, and *Ppp1r15a*) were identified as the hub genes. **(B)** Network of hub genes. **(C)** Information on six hub genes. **(D)** Heatmap of 174 ferroptosis-related genes. **(E)** Venn diagram showing nine ferroptosis-related DEGs. Red circle represents 207 DEGs between groups, blue circle represents 174 ferroptosis-related genes, and the overlap represents nine ferroptosis-related DEGs. **(F)** Information about nine ferroptosis-related DEGs.

To explore whether ferroptosis is involved in OGDR-induced cell death, we identified ferroptosis-related DEGs. We downloaded the ferroptosis dataset from NCBI and obtained 174 ferroptosis-related genes by comparing the ferroptosis and RNA-seq datasets. The differential distribution of 174 ferroptosis-related genes between the samples is shown in a heatmap (Figure 3C). Using Venn diagram analysis (Figure 3D), nine ferroptosis-related DEGs were identified, comprising two upregulated genes (*Ccn2 and Tfrc*) and seven downregulated genes (*Atf3*, *Slc7a11*, *Slc3a2*, *Chac1*, *Gch1*, *Lcn2*, and *Sesn2*) (Figure 3F).

To further explore the relationship between ferroptosis and OGDR, this study focused on key subnetworks and hub genes. We constructed a PPI network of 207 DEGs, which included 113 nodes and 207 edges. Combined with the MCODE plugin, the top1 cluster with the highest score was obtained, and six genes in the cluster were identified as hub genes: *Ddit3*, *Atf3*, *Sesn2*, *Trib3*, *Gadd45a*, and *Ppp1r15a* (Figures 3A, B). The expression of six hub genes was downregulated in the OGDR group compared to the control group (Figure 3E). Interestingly, *Atf3* and *Sesn2* were both hub genes and ferroptosis-related DEGs.

**FIGURE 4:**
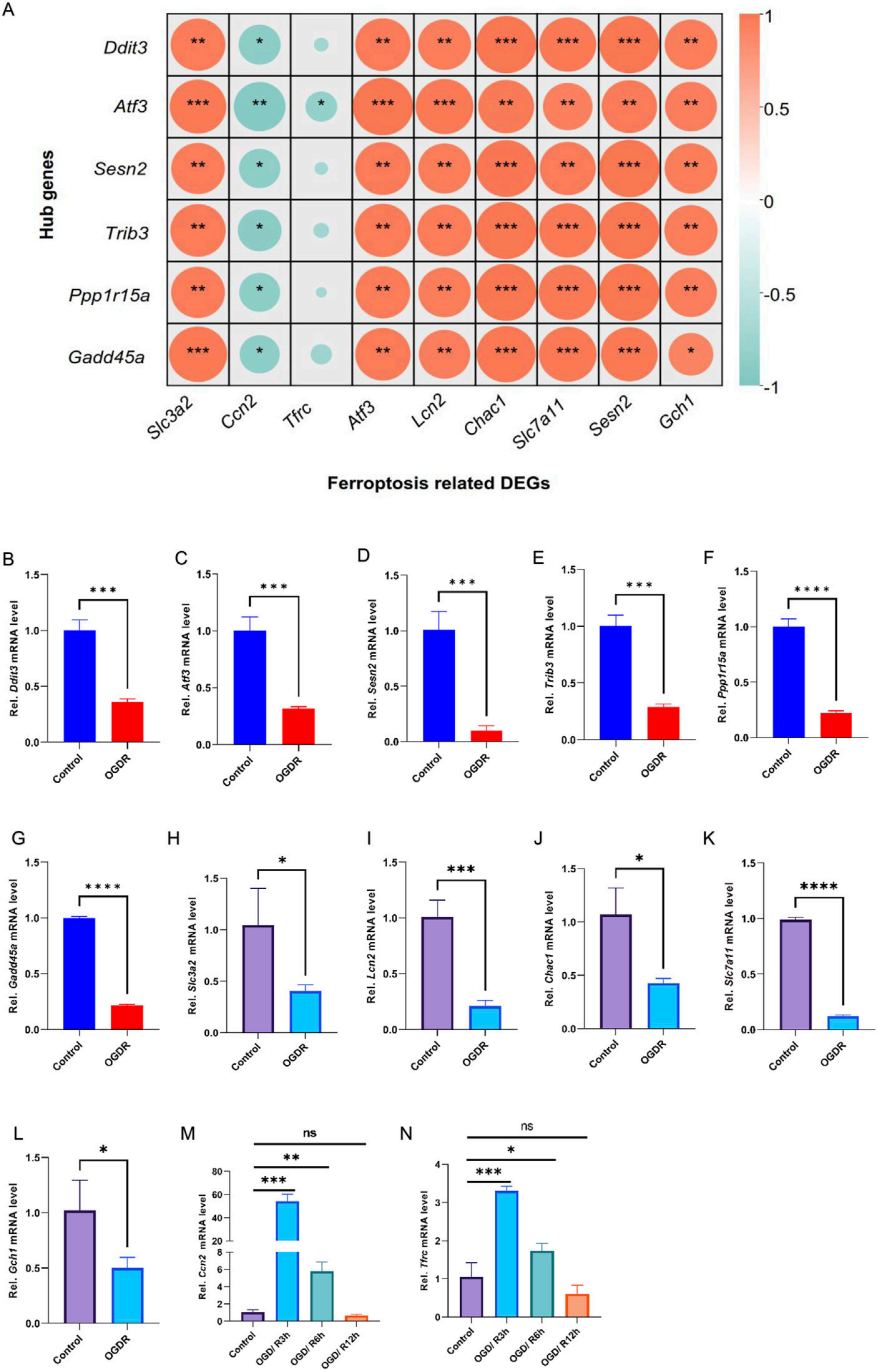
The correlation showed a significant correlation between the hub genes and ferroptosis-related DEGs, and their mRNA expression levels were verified by RT-qPCR. **(A)** Heatmap of the Spearman correlation between hub genes and ferroptosis-related DEGs by ChiPlot. Different colors represent different correlations (red represents a positive correlation, and blue represents a negative correlation). The circle size represents the correlation coefficient (the larger circle represents stronger correlation, and the smaller circle represents a weaker or less correlation). *p < 0.05; **p < 0.01; and ***p < 0.001. **(B–N)** Validation of hub genes and ferroptosis-related DEGs by RT-qPCR. **(B–G)** mRNA expression levels of *Ddit3*, *Atf3*, *Sesn2*, *Trib3*, *Gadd45a*, and *Ppp1r15a*. The mRNA expression trends of six hub genes were consistent with the RNA-seq results. **(H–N)** The mRNA expression levels of *Slc3a2*, *Lcn2*, *Chac1*, *Slc7a11*, *Gch1*, *Ccn2*, and *Tfrc* were analyzed. A total of nine ferroptosis-related DEGs showed the same trend as the results of RNA-seq.

RNA-seq data showed that the expression of hub genes was significantly different between the control and OGDR groups. To further verify the reliability of these data, we evaluated the mRNA expression levels of six hub genes (Table 4) using RT-qPCR. The results showed that the mRNA expression levels of all hub genes, *Ddit3*, *Atf3*, *Sesn2*, *Trib3*, *Gadd45a*, and *Ppp1r15a*, in the OGDR group were significantly downregulated compared to those in the control group (Figures 4B–G), which was consistent with the RNA-seq results.

**TABLE 4:** Features and functions of six hub genes.

Gene	Full name	Function	References
Atf3	Activating transcription factor 3	This protein binds the cAMP response element (CRE), a sequence present in many viral and cellular promoters and represses transcription from promoters with ATF sites. It belongs to the bZIP family, ATF subfamily	ATF3 promotes erastin-induced ferroptosis through the suppressing system Xc− (Wang et al., 2019)
Gadd45a	Growth arrest and DNA damage-inducible protein GADD45 alpha	Might affect PCNA interactions with some CDK (cell division protein kinase) complexes, stimulates DNA excision repair *in vitro*, and inhibits entry of cells into the S phase. Belongs to the GADD45 family	GADD45A inhibits autophagy by regulating the interaction between BECN1 and PIK3C3 (Zhang et al., 2016)
SESN2	Sestrin-2	Functions as an intracellular leucine sensor that negatively regulates the TORC1 signaling pathway through the GATOR complex. This stress-inducible metabolic regulator also plays a role in protection against oxidative and genotoxic stresses	SESN2 can act as a potential regulator of mitochondrial quality control, following induction by ROS under stress conditions (Piochi et al., 2021)
Ddit3	DNA damage-inducible transcript 3 protein	Multifunctional transcription factor in ER stress response. Plays an essential role in the response to a wide variety of cell stresses and induces cell cycle arrest and apoptosis in response to ER stress	DDIT3 is induced during glutamine deprivation and dampens the sustained levels of reactive oxygen species (Li et al., 2021b)
Trib3	Tribble homolog 3	Disrupts insulin signaling by binding directly to Akt kinases and blocking their activation. Binds to ATF4 and inhibits its transcriptional activation activity. ATF4 and CHOP bind to Trib3 promoter to activate Trib3 transcription	Trib3 is induced in neurons in response to ferroptotic stimuli via an ATF4-dependent pathway (Ratan, 2020)
Ppp1r15a	Protein phosphatase 1 regulatory subunit 15A	Recruits the serine/threonine-protein phosphatase PP1 to dephosphorylate the translation initiation factor eIF-2A/EIF2S1, thereby reversing the shut-off of protein synthesis initiated by stress-inducible kinases and facilitating recovery of cells from stress	Ppp1r15a as a stress-inducible regulatory subunit recruits PP1 to dephosphorylate eIF2α (Carrara et al., 2017)

The mRNA expression levels of nine ferroptosis-related DEGs were verified using RT-qPCR. The mRNA levels of *Atf3*, *Sesn2*, *Slc3a2*, *Lcn2*, *Chac1*, *Slc7a11*, and *Gch1* were significantly downregulated (Figures 4C, D, 4H–L), consistent with the RNA-seq results. The expression of *Ccn2* and *Tfrc* was upregulated in RNA-seq analysis, and the results of RT-qPCR also verified this trend. After reoxygenation and re-glucose, the expression of *Ccn2* and *Tfrc* was significantly upregulated at R3h and R6h, although there was no significant difference in expression at R12h (OGDR group), compared with control group (Figures 4M, N).

### 3.4 Correlation analysis showed a significant correlation between ferroptosis-related DEGs and hub genes

To evaluate the role of ferroptosis in the OGDR model, Spearman’s correlation analysis was performed between the six hub genes and nine ferroptosis-related DEGs. As predicted, there was a significant correlation between the hub genes and ferroptosis-related DEGs. Seven ferroptosis-related DEGs, namely, *Atf3*, *Slc7a11*, *Slc3a2*, *Chac1*, *Gch1*, *Lcn2*, and Sesn2, were significantly positively correlated with six hub genes, whereas *Ccn2* was significantly negatively correlated with six hub genes, and *Tfrc* was significantly negatively correlated with the hub gene *Atf3* (Figure 4A).

### 3.5 Ferroptosis was involved in OGDR/IR injury

**FIGURE 5:**
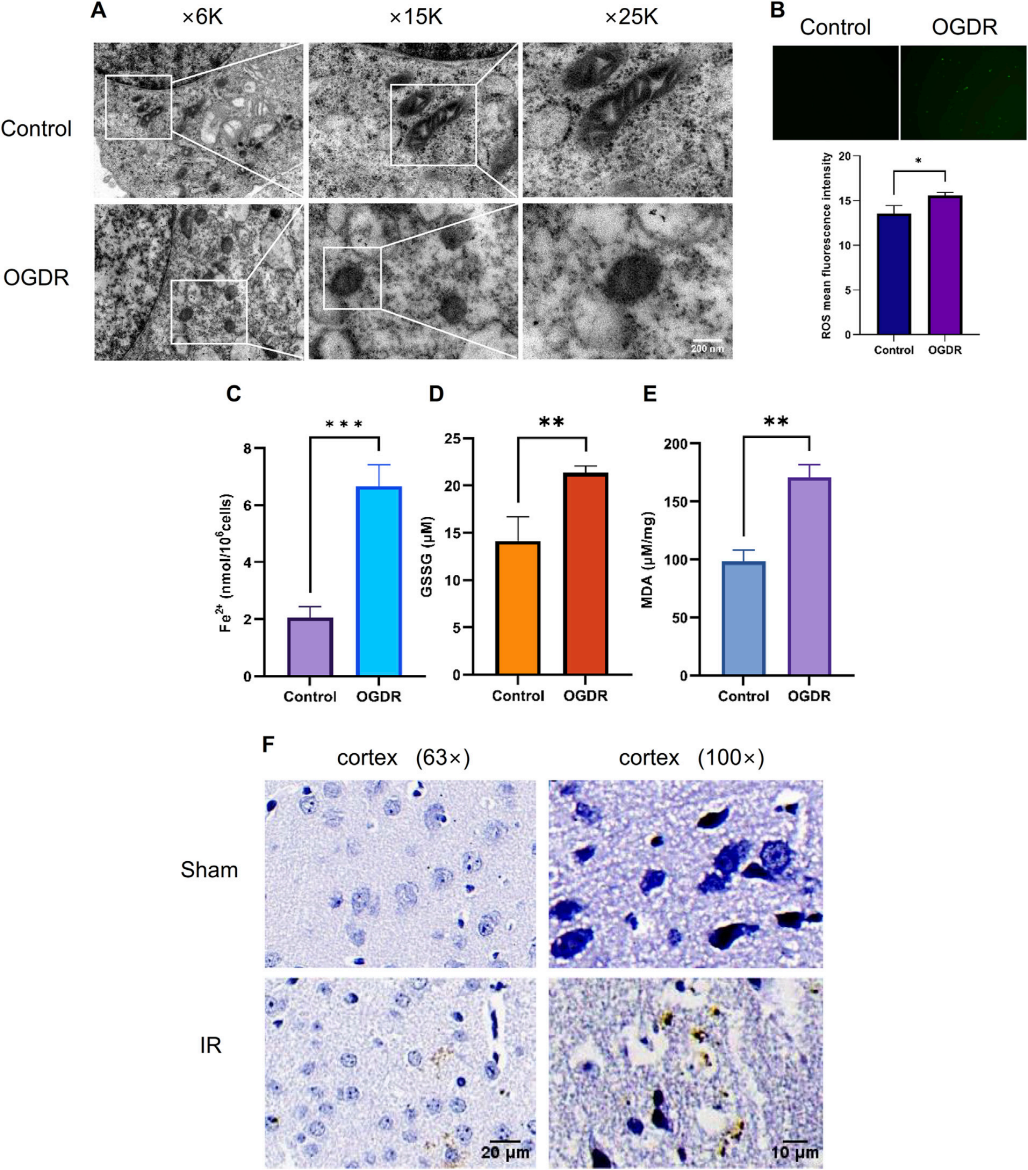
Ferroptosis was involved in OGDR/IR injury. **(A)** Representative TEM images of the bEND.3 cells in the control and OGDR groups at different magnifications: mitochondrial shrinkage, density increases, mitochondrial crest disappears, and mitochondrial membrane ruptures. **(B–E)** Detection of ROS, Fe^2+^, GSSG, and MDA in bEND.3 cell samples. **(B)** Fluorescence detection of ROS. No obvious green fluorescent signal was detected in the control group. More green fluorescence signals were detected in the OGDR group. **(C)** bEND.3 cell Fe^2+^ content. **(D)** bEND.3 cell GSSG content. **(E)** bEND.3 cell MDA content. **(F)** Representative Prussian blue staining images of the cerebral cortex in the Sham group and IR group at different magnifications. Significant iron deposition (brown color) was observed in the IR group.

To verify OGDR-induced ferroptosis, mitochondrial morphology was observed using TEM, and the levels of ROS, Fe^2+^, GSSG, and MDA were measured. TEM images revealed the classic mitochondrial morphological changes in ferroptosis: mitochondrial shrinkage, density increase, mitochondrial crest disappearance, and mitochondrial membrane rupture in bEND.3 cells after OGDR treatment (Figure 5A). The levels of ROS, Fe^2+^, GSSG, and MDA in the OGDR-treated bEND.3 cells were significantly higher than those in the control group (Figures 5B–E). *In vivo*, Prussian blue staining showed significant iron deposition in the cerebral cortex, following IR injury (Figure 5F). These results suggested that OGDR/IR induces ferroptosis.

### 3.6 GPX4, a marker protein of ferroptosis, was reduced after OGDR, and Fer-1, an inhibitor of ferroptosis, partly restored the level of GPX4

**FIGURE 6:**
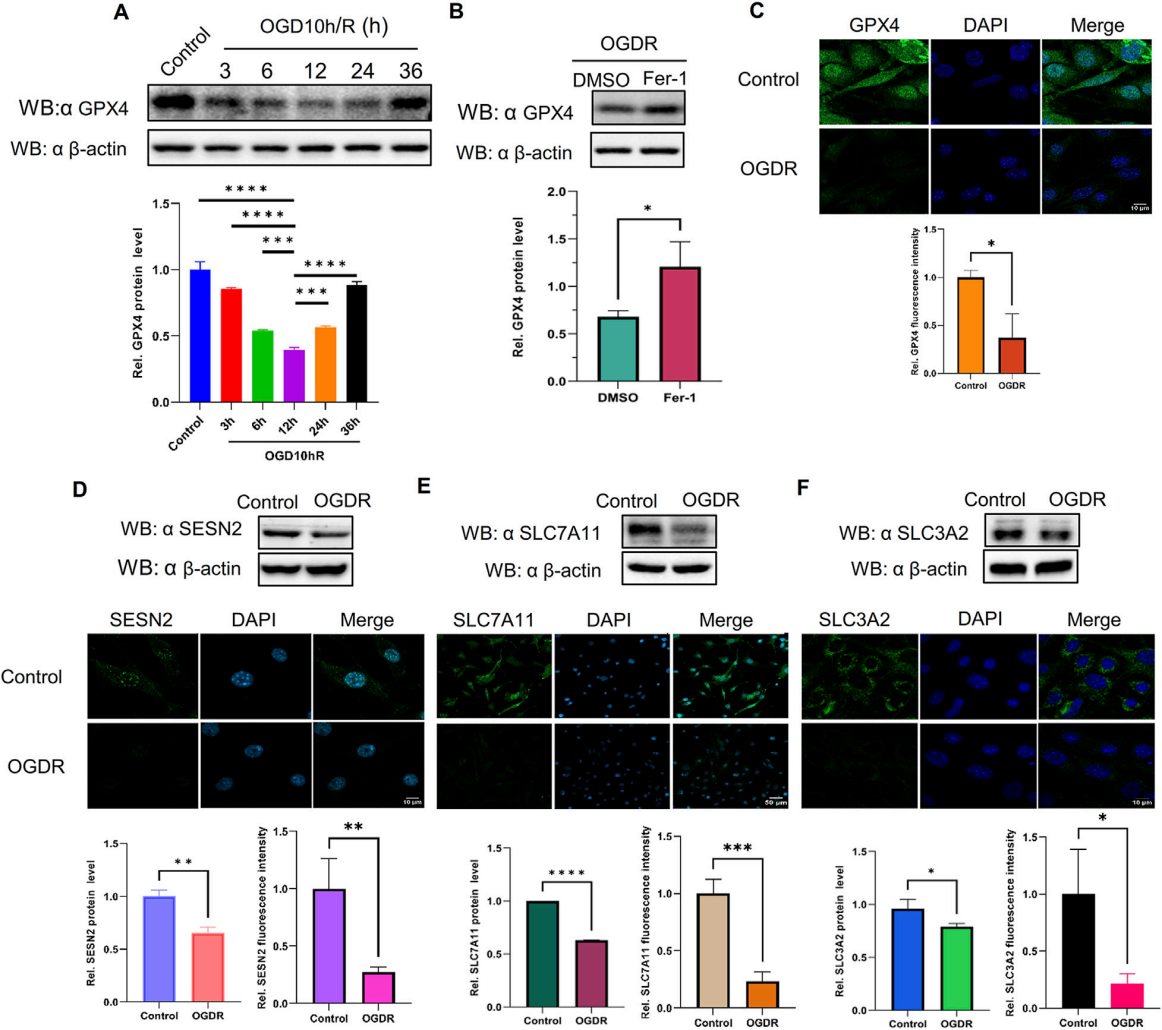
The protein expression levels of GPX4, SESN2, and System Xc− were analyzed by western blot and immunofluorescence in bEND.3 cells. Green fluorescence represents GPX4, SESN2, SLC7A11, or SLC3A2. Blue fluorescence labels the nucleus. **(A)** The expression level of GPX4 was significantly decreased during reoxygenation and re–glucose deprivation and reached the lowest at R12h. **(B)** Fer-1 pretreatment partly restored the expression of GPX4. **(C)** GPX4 expression was also analyzed by immunofluorescence. **(D)** Compared with the control group, the protein expression of SESN2 was significantly reduced in the OGDR group. **(E–F)** The ferroptosis-related System Xc− proteins SLC7A11 **(E)**, and SLC3A2 **(F)** were analyzed by western blot and immunofluorescence.

**FIGURE 7:**
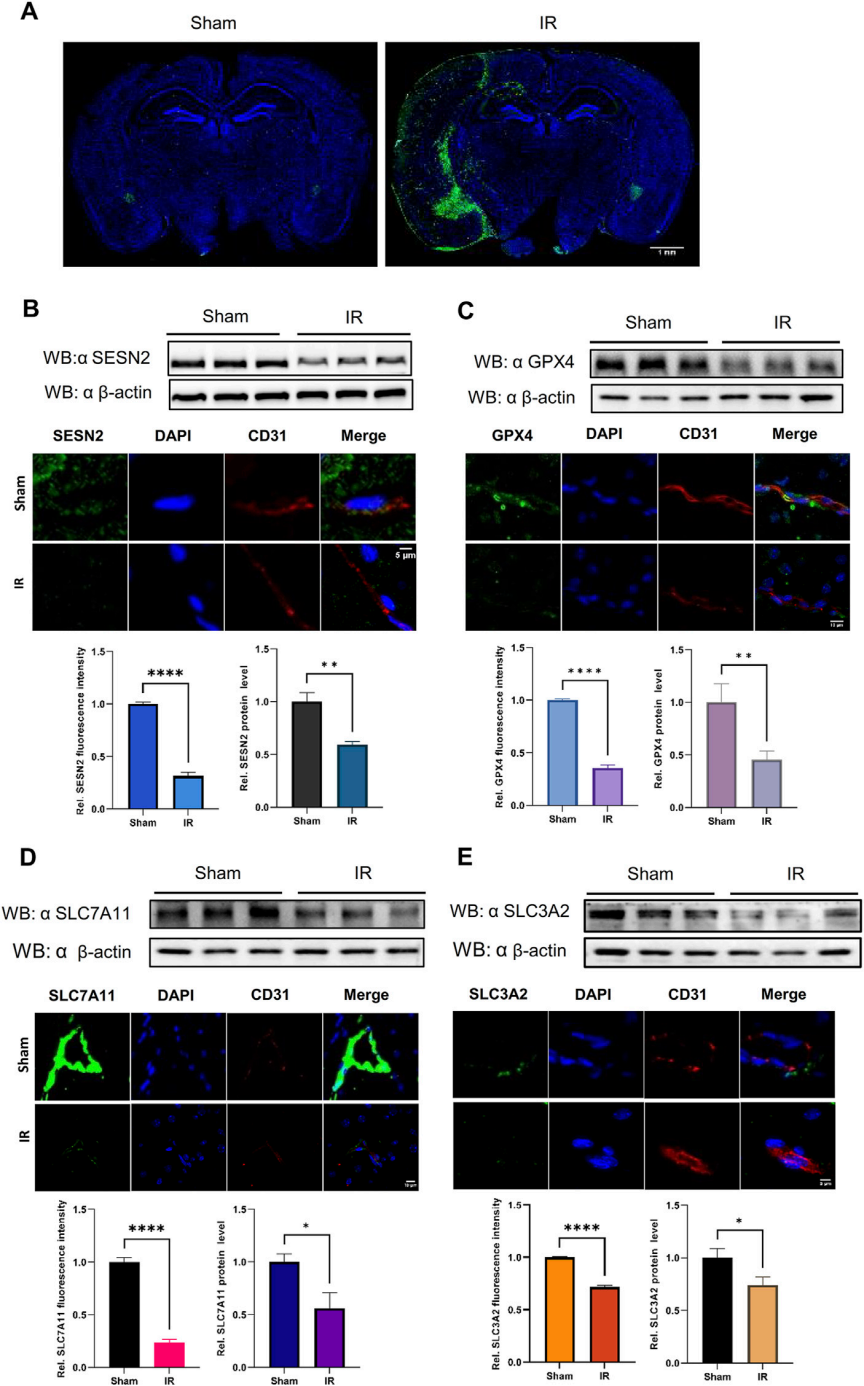
The protein expression levels of SESN2 and ferroptosis-related proteins were analyzed by western blot and immunofluorescence in the cerebral cortex of IR. Green fluorescence represents HMOX1, SESN2, GPX4, SLC7A11, or SLC3A2. Red fluorescence labels microvascular endothelial cells. Blue fluorescence labels the nucleus. **(A)** Representative immunofluorescence staining of the mouse brain in the Sham and IR groups: HMOX1 (green) and nucleus (blue). **(B)** Compared with the Sham group, the protein expression of SESN2 was significantly reduced in the IR group. **(C–E)** The protein expression levels of GPX4 **(C)**, SLC7A11 **(D)**, and SLC3A2 **(E)** were analyzed by western blot and immunofluorescence after IR and showed a trend consistent with OGDR-treatment bEND.3 cells.

The expression of GPX4, a well-known ferroptosis-negative regulatory protein, was detected at different time points by western blot. The results showed that GPX4 expression after OGDR treatment decreased, reached its lowest at R12h, and then gradually resumed (Figure 6A). Immunofluorescence assays also demonstrated a reduction in OGDR-induced GPX4 expression (Figures 6C, 7C). Furthermore, pretreatment with Fer-1 (a small-molecular inhibitor of ferroptosis) alleviated the downregulation of GPX4 expression at R12h (Figure 6B).

### 3.7 SESN2 and system Xc− proteins were downregulated induced following OGDR/IR injury

To ascertain the expression of SESN2 protein more clearly, the focal cerebral IR model was establised (Figure 7A), and we collected samples from cells and brain tissues and measured the protein expression of SESN2 by western blot and immunofluorescence. As shown in Figures 6D, 7B, SESN2 protein expression was downregulated by OGDR/IR injury. Furthermore, we examined the expressions of SLC7A11 and SLC3A2, two component proteins of System Xc−. The expression of both SLC7A11 (Figures 6E, 7D) and SLC3A2 (Figures 6F, 7E) decreased after OGDR/IR treatment.

### 3.8 SESN2 negatively regulated OGDR-induced ferroptosis *in vitro* through the system Xc−/GPX4 pathway

**FIGURE 8:**
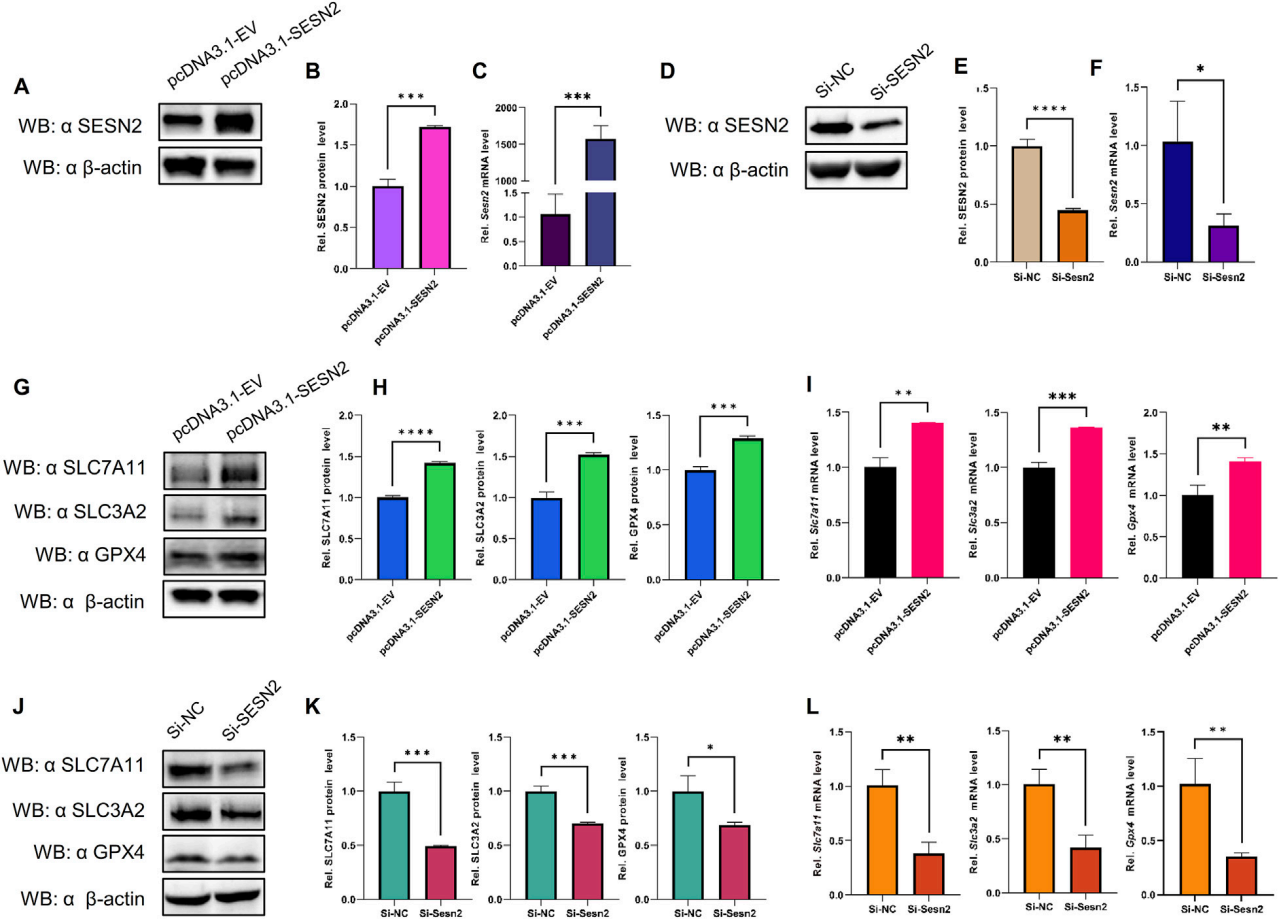
SESN2 was involved in OGDR-induced ferroptosis by regulating the System Xc−/GPX4 pathway. **(A–C)** Transfection efficiency of plasmid pcDNA3.1-SESN2 was verified by western blot **(A, B)** and RT-qPCR **(C)**. **(D–F)** Interference efficiency of siRNA was verified by western blot **(D–E)** and RT-qPCR **(F)**. **(G–I)** SESN*2* overexpression upregulated the protein **(G, H)** and mRNA expression levels **(I)** of SLC7A11, SLC3A2, and GPX4. **(J–L)** SESN2 interference decreased the protein **(J–K)** and mRNA expression levels **(L)** of SLC7A11, SLC3A2, and GPX4.

To examine the relationship between SESN2 and ferroptosis, an SESN2 overexpression plasmid was transfected into bEND.3 cells, and siRNA was used to silence SESN2. Overexpression (Figures 8A–C) and knockdown (Figures 8D–F) efficiency were validated using western blot and RT-qPCR.

The results revealed that SESN2 overexpression significantly increased the expression of GPX4 compared to the empty-vehicle group (Figures 8G–I), whereas SESN2 suppression significantly decreased the expression of GPX4 compared to the negative control group (Figures 8J–L). These findings suggested that SESN2 protects bEND.3 cells against OGDR-induced ferroptosis in bEND.3 cells.

To explore the impact of SESN2 on ferroptosis more clearly, we examined the changes in the crucial molecules of ferroptosis in response to SESN2 overexpression and silencing. The results showed that overexpression of SESN2 attenuated the OGDR-induced downregulation of SLC7A11 and SLC3A2 (Figures 8G–I). Conversely, the knockdown of SESN2 decreased the expression of SLC7A11 and SLC3A2 (Figures 8J–L). These results revealed that SESN2 negatively regulated OGDR-induced ferroptosis in bEND.3 cells through the System Xc−/GPX4 pathway.

## 4 Discussion

Ischemia and reperfusion can cause secondary brain injury with two major manifestations, namely, symptomatic hemorrhagic transformation and malignant edema, which are intractable and catastrophic. BBB degradation is a mechanism underlying secondary injury after reperfusion (Campbell and Khatri, 2020). Brain microvascular endothelial cells, key components of the BBB, are the first line of defense in the BBB, playing a crucial role in maintaining brain homeostasis (Segarra et al., 2021). Previous studies on brain microvascular endothelial cells have mainly focused on tight junction structural complexes, angiogenesis, and drug delivery (Feng et al., 2018; Li et al., 2022c; Yang et al., 2020). This study focused on BMVECs ferroptosis after reperfusion.

Ferroptosis, a key driver of IR injury and organ failure, has been reported to be involved in IR injury (Li X. et al., 2021; Wang et al., 2021; Cai et al., 2023; Li et al., 2022d; Liu et al., 2022). During reperfusion, the accumulation of excessive ROS in cells, membrane structure lipid peroxidation, and intracellular iron overload leads to ferroptosis (Li X. et al., 2021). Correspondingly, several studies on heart IR injury have demonstrated that ferroptosis occurs mainly during the reperfusion period rather than the ischemic period (Tang et al., 2021), and it is considered the predominant form of cell death during prolonged reperfusion (Cai et al., 2023). In IR injury of numerous organs (Li X. et al., 2021; Luan et al., 2024), including the heart (Cai et al., 2023; Tang et al., 2021; Liu et al., 2022), brain (Zhu et al., 2022; Xu et al., 2023; Xu et al., 2023; Yuan et al., 2021), kidney (Li et al., 2022d; Zhao et al., 2020), liver (Li C. et al., 2023), intestine (Xu et al., 2021a; Chu et al., 2023; Chu et al., 2023), and lung (Wang et al., 2023a), ferroptosis has been shown to play a crucial role, and its underlying regulatory mechanisms have also been continuously elucidated. However, the effect of ferroptosis on BMVECs injury after reperfusion remains unclear. In this study, RNA-seq, bioinformatic analysis, and systematic biological approaches were used. The results showed that endothelial cell damage induced by OGDR/IR is closely related to ferroptosis and that SESN2 is an important regulator of ferroptosis. This study is invaluable for further understanding of the mechanisms underlying brain IR injury.

In this study, the bEND.3 cell line, commonly used in blood–brain barrier research, was treated with OGDR to mimic IR injury. RNA-seq was performed, and the differential expression of transcriptional levels induced by OGDR was analyzed using bioinformatics. The results showed that 207 DEGs were mainly enriched in “cellular process, cell growth and death, amino acid metabolism, lipid metabolism, and glutathione metabolic process” based on GO term, KEGG, and Reactome pathway analysis. These pathways are associated with ferroptosis (Stockwell et al., 2017; Chen et al., 2020). As is widely agreed, amino acid metabolism plays an important role in the occurrence of ferroptosis. For example, the cysteine–glutathione axis is one of the most important amino acid metabolic pathways regulating ferroptosis. Glutathione is synthesized from cysteine and negatively regulates ferroptosis. In a pancreatic tumor study, the deletion of cysteine induced ferroptosis in pancreatic ductal adenocarcinoma (Badgley et al., 2020). Lipid metabolism profoundly affects ferroptosis by regulating phospholipid peroxidation. Targeting key molecules that mediate lipid peroxidation can be used in cancer therapy to induce ferroptosis (Dodson et al., 2019).

Based on the identified 207 DEGs, we constructed a PPI network to predict hub genes using MCODE component analysis. Finally, six genes in the cluster with the highest scores were identified as hub genes, namely, *Ddit3*, *Atf3*, *Sesn2*, *Trib3*, *Gadd45a*, and *Ppp1r15a*. Spearman’s correlation analysis showed that hub genes were significantly associated with ferroptosis-related DEGs. These correlation results provide further theoretical support for the induction of ferroptosis by ODGR, which may be an important type of OGDR-induced cell death.

Atf3, a common stress sensor, bound to the Slc7a11 promoter, suppressed the expression of System Xc−, and promoted ferroptosis (Feng et al., 2018; Wang Y. et al., 2022; Lu et al., 2021). Interestingly, a study on myocardial IR reported the opposite: Atf3 inhibits cardiomyocyte ferroptosis after IR by regulating FANCD2, and Atf3 knockout significantly aggravated IR injury (Lu et al., 2021). These results support those of our study, in which reperfusion and ferroptosis were involved and the mRNA expression of *Atf3* was significantly downregulated. SESN2, a highly evolutionary and stress-responsive protein, has recently been shown to affect the pathological processes of ferroptosis. Studies have indicated that SESN2 can suppress ferroptosis of DCs in sepsis by downregulating the Atf4/Chop/Chac1 signaling pathway (Li J.-Y. et al., 2021), and modulating the SESN2/AMPK/Nrf2/HO-1 signaling pathway can mitigate ferroptosis in pulmonary fibrosis (El-Horany et al., 2023). In the abovementioned studies (Li J.-Y. et al., 2021; El-Horany et al., 2023), SESN2 was considered a negative regulator of ferroptosis, which agrees with the downregulated expression of SESN2 in our ferroptosis study. As a pro-death protein, Trib3 is induced in neurons in response to ferroptosis via the Atf4-dependent pathway (Ratan, 2020). In a non-ferroptotic study, Trib3 deletion was found to reduce cell death in a mouse model of global ischemia (Wei et al., 2017). However, the role of Trib3 in the regulation of ferroptosis remains unclear and needs further clarification. Ddit3, Ppp1r15a, and Gadd45a belong to the growth arrest and DNA damage families, are induced by multiple stresses, and produce ROS. Ddit3, also known as growth arrest and DNA damage 153 (Gadd153) or C/EBP homologous protein (CHOP), is a transcription factor (Li M. et al., 2021). Reports have shown that Ddit3 is involved in ferroptosis-induced endoplasmic reticulum stress (Lee et al., 2018) and that Ddit3 is downregulated by SESN2 to suppress ferroptosis (Li J.-Y. et al., 2021). However, Ddit3 expression was downregulated in the OGDR group. Ppp1r15a, also known as growth arrest and DNA damage-inducible protein (Gadd34), was highly expressed under the stimulation of acrolein and in response to ROS production (Sun et al., 2015). Gadd45a, a Tp53-regulated and DNA-damage-responsive protein, induces cell cycle arrest, apoptosis, DNA damage repair, and angiogenesis inhibition (Zhang et al., 2016). To date, there has been limited research on the relationship between Ppp1r15a/Gadd45a and ferroptosis, which presents potential areas for further exploration. Gene regulation is an extremely complex process influenced by many factors, including the species, model, treatment, time, and space, which together determine similar or opposite regulatory outcomes. In addition, the final contribution values (protein levels) of the genes were different, which may explain the contradictory results. The mRNA expression levels of the six hub genes were verified by RT-qPCR, and the results were consistent with the RNA-seq results.

To highlight that ferroptosis is associated with OGDR/IR, we tested biochemical markers of ferroptosis. Levels of ROS, Fe^2+^, GSSG, and MDA were markedly higher in the OGDR group, supporting the ferroptosis hypothesis. Typical ferroptotic mitochondrial morphological changes were observed by TEM. Immunohistochemistry of brain sections revealed significant iron deposition in reperfusion cortical areas. Several key ferroptosis-related proteins were also examined. GPX4 is believed to be the central point against ferroptosis and is considered a marker of ferroptosis. GPX4 catalyzes the reduction of toxic lipid hydroperoxides (L-OOH) to non-toxic alcohols (L-OH), whereas oxidation reduces glutathione (GSH) to oxidized glutathione (GSSG) (Costa et al., 2023). Thus, GPX4 inactivation leads to the accumulation of lipid peroxides and promotes ferroptosis (Costa et al., 2023; Chen et al., 2020). The downregulated expression of GPX4 induced by OGDR/IR was validated by western blot and immunofluorescence. Fer-1, a ferroptosis inhibitor, partially counteracted this decline. System Xc−, a membrane bidirectional transporter, exchanges intracellular glutamate and extracellular cysteine to participate in the generation of glutathione and regulate the redox state, preventing the ferroptosis of cells (Liu et al., 2021). System Xc− is composed of two subunits, SLC7A11 and SLC3A2. SLC7A11 provides a transport function and is considered the core component of System Xc−. SLC3A2 is critical for the maintenance of structural stability. System Xc−/GPX4 is an important ferroptosis regulatory pathway, and the inhibition of System Xc− could promote the occurrence of ferroptosis (Wang et al., 2019). In our study, the protein expression of SLC7A11 and SLC3A2 was significantly downregulated in the OGDR and IR groups, which was consistent with the RNA-seq transcriptional-level analysis. The altered expression of these key proteins provides further evidence of ferroptosis.

In this study, SESN2, a hub gene, was downregulated at both the transcriptional and translational levels after OGDR/IR. Consistent with our findings, studies on IR injury in the brain (Liu et al., 2020), intestine (Zhang L. L. et al., 2024), and heart (Zhou X. R. et al., 2021) also confirmed that SESN2 expression is downregulated by reperfusion and exacerbates tissue oxidative stress damage. Interestingly, some studies have shown that SESN2 expression is increased after IR in the brain (Yang et al., 2023; Li et al., 2016), heart (Li et al., 2024), and kidney (Ishihara et al., 2013) and plays a protective role by reducing mitochondrial stress, regulating autophagy, and inhibiting ferroptosis. These differences suggest that the responses of SESN2 cells to IR injury may be tissue- or cell-specific or influenced by different experimental conditions. However, SESN2 is a protective regulator of IR injury, and as previously mentioned, SESN2 has an inhibitory effect on ferroptosis, thereby alleviating the pathological process of diseases (Yang et al., 2023; Liu et al., 2020; Li et al., 2016; Li et al., 2024; Ishihara et al., 2013). To determine whether there has been a regulatory effect of SESN2 on ferroptosis, SESN2 was knocked down and overexpressed *in vitro*. First, we tested the expression of the ferroptosis marker protein, GPX4, to demonstrate the regulatory effect of SESN2 on ferroptosis. The expression of the GPX4 upstream regulators, SLC7A11 and SLC3A2, was analyzed to verify the regulatory pathways. Upon silencing SESN2, GPX4 was downregulated at both mRNA and protein levels. Conversely, SESN2 overexpression increased GPX4 expression. SLC7A11 and SLC3A2 expression was reduced by SESN2 silencing or promoted by SESN2 overexpression. The System Xc−/GPX4 axis is an important antioxidant system that plays a key role in preventing ferroptosis (Li F. J. et al., 2022). Studies have shown that repression of the System Xc−/GPX4 axis drives ferroptosis of vascular smooth muscle cells (Ye et al., 2022), whereas naringenin alleviates myocardial IR injury by activating the system Xc−/GPX4 axis to inhibit ferroptosis (Xu et al., 2021b). These findings suggested that SESN2 negatively regulated OGDR-induced ferroptosis in bEND.3 cells by inhibiting the System Xc−/GPX4 pathway.

Several studies have reported an association between brain microvascular endothelial cells and ferroptosis (Wang P. et al., 2022; Liu et al., 2023; Li P. et al., 2023). Liu et al. (2023) demonstrated that ferroptosis is involved in bEND.3 cells injury in the OGD model, but their study did not implement reoxygenation or RNA-seq analysis, which our research highlights. Wang P. et al. (2022) found that iron-mediated oxidative stress is an early cause of brain microvascular endothelial cell damage after IR and that overexpression of FtMt attenuated IR-induced BBB disruption. Perhaps, due to the different models used, FtMt mRNA was not detected in the present study. Based on RNA-seq and bioinformatics analyses, our study demonstrated that reperfusion induces ferroptosis in BMVECs, and preliminary mechanistic studies have been conducted. Future studies should explore the molecular mechanisms underlying ferroptosis based on the hub genes. We set relatively strict differential gene screening criteria, which may have resulted in the omission of genes with large actual contributions. From the table of reads distribution statistics in different regions, we can observe that more reads are distributed in the 3′UTR region, suggesting that transcriptome epigenetic modifications may play an important regulatory function, and we should pay attention to it in future research.

In summary, using RNA-seq and bioinformatics analysis, this study systematically analyzed the enrichment and interaction of DEGs in bEND.3 cells induced by OGDR and identified six ferroptosis-related hub genes. Bioinformatics results suggest that ferroptosis plays a crucial role in OGDR, which has been verified successfully. In this study, the SESN2/System Xc−/GPX4 pathway negatively regulated reperfusion-induced ferroptosis. The current research results will contribute to the future mechanistic exploration of IR-induced ferroptosis in BMVECs and provide a theoretical basis for the treatment of IR injury and the development of targeted drugs.

## Data Availability

The data presented in the study are deposited in the NCBI BioProject repository, accession number PRJNA1065615. The data deposition can be find in the following link: https://www.ncbi.nlm.nih.gov/bioproject/PRJNA1065615/.
